# Angular Motion Estimation Using Dynamic Models in a Gyro-Free Inertial Measurement Unit

**DOI:** 10.3390/s120505310

**Published:** 2012-04-26

**Authors:** Ezzaldeen Edwan, Stefan Knedlik, Otmar Loffeld

**Affiliations:** 1 Center for Sensor Systems (ZESS), University of Siegen, Paul Bonatz-Str. 9-11, 57068 Siegen, Germany; E-Mail: loffeld@zess.uni-siegen.de; 2 iMAR GmbH, St. Ingbert, Germany; E-Mail: s.knedlik@imar-navigation.de

**Keywords:** angular motion estimation, GF-IMU, dynamic models

## Abstract

In this paper, we summarize the results of using dynamic models borrowed from tracking theory in describing the time evolution of the state vector to have an estimate of the angular motion in a gyro-free inertial measurement unit (GF-IMU). The GF-IMU is a special type inertial measurement unit (IMU) that uses only a set of accelerometers in inferring the angular motion. Using distributed accelerometers, we get an angular information vector (AIV) composed of angular acceleration and quadratic angular velocity terms. We use a Kalman filter approach to estimate the angular velocity vector since it is not expressed explicitly within the AIV. The bias parameters inherent in the accelerometers measurements' produce a biased AIV and hence the AIV bias parameters are estimated within an augmented state vector. Using dynamic models, the appended bias parameters of the AIV become observable and hence we can have unbiased angular motion estimate. Moreover, a good model is required to extract the maximum amount of information from the observation. Observability analysis is done to determine the conditions for having an observable state space model. For higher grades of accelerometers and under relatively higher sampling frequency, the error of accelerometer measurements is dominated by the noise error. Consequently, simulations are conducted on two models, one has bias parameters appended in the state space model and the other is a reduced model without bias parameters.

## 1. Introduction

A conventional IMU is composed of three accelerometers and three gyroscopes mounted in a strap-down configuration. Accelerometers are sensors that measure acceleration and gyroscopes are sensors that measure the angular rate of rotation. Gyros are usually corrupted by various sources of errors such as bias instability, noise, scale factor errors… *etc*. For gyros, the bias instability is the dominant error because the inertial system often works as a standalone system. Therefore, inertial systems can be categorized in terms of gyro bias error [1]. It is shown in [1,2] that the biases of the gyros play an important role in causing drift in the position by an example of a biased gyro which causes an error in the position that grows with the cube of time.

The use of distributed accelerometers as an alternative to conventional gyros to infer the angular motion has been a subject of intensive research. Unlike the standard IMU, the GF-IMU uses only accelerometers to infer the acceleration and the angular velocity. It is possible to get the Coriolis acceleration vector, which contains a direct expression of the angular velocity vector, through configurations contain rotating accelerometers. However, the focus of this work is on fixed accelerometer configurations because they are simpler to implement. There are several reasons to use accelerometers for inferring the angular motion. Generally, accelerometers are less costly, less heavy and less power consuming than comparable gyros, which have typically the disadvantage of complicated manufacturing techniques, high cost, high power consumption, high weight, large volume, and limited dynamic range [3]. A GF-IMU can be used to measure the angular velocities in crashworthiness, sports and motion analysis applications, which are characterized by large peak values of the angular velocity as listed in [4]. A survey of the GF-IMU literature and its research areas can be found in [5].

The rest of this paper is organized as follows: Section 2 gives a background about the angular motion estimation in a GF-IMU and describes the configuration used in this work. Section 3 lists the dynamic models which can be used for the Kalman filter process update. Section 4 gives a sensor error model with a review of the calibration procedure. Section 5 presents an extended Kalman filter (EKF) solution using a Singer model with appended bias parameters. Section 6 presents the observability analysis for the augmented state space model. Section 7 presents an EKF solution without appending bias parameters. Section 8 gives simulation results for the augmented model and Section 9 gives simulation results for the reduced model. Finally, Section 10 presents our conclusions.

## 2. Angular Motion Estimation in a GF-IMU

Using certain fixed GF-IMU configurations of accelerometers, we get an angular acceleration vector and quadratic terms of the angular velocity. Quadratic angular velocity terms do not have an accumulative error as in the case when the angular acceleration is integrated. Proper filter setup combining the angular acceleration and the quadratic terms can assist in the convergence to the right sign as the quadratic terms have undetermined sign solutions. The integration of the different types of data coming from the GF-IMU has been a subject of intensive research. In [5], an EKF solution using direct three state models based on Euler first order integration is given. The advantage of using such a model is that no assumption needs to be made about the dynamics of the motion and hence such a solution fits most scenarios. Reference [6] gives a nine state model which includes the angular acceleration, the angular velocity and the translational acceleration vectors. The solution for that model uses an unscented Kalman filter (UKF) with constant angular acceleration and translational acceleration models and without appending the bias parameters. An EKF solution with measurement vector which uses three quadratic velocity terms measurements is given in [7] for a configuration of nine fixed accelerometers. However, that GF-IMU configuration produces three quadratic terms besides the angular acceleration vector. We use dynamic models to describe the evolution of the state space in time to have an estimate of the angular motion. Bias parameters can be appended to the state vector if their estimation is desired. Though the first order Euler integration model has a simple form, some of the bias parameters, which exist in most of the inertial sensors, will not be observable. Hence, it fits cases where white noise is corrupting accelerometers measurements. The importance of this research is that within a proper dynamic model, the drifting biases can be estimated and hence the quality of the estimated angular velocity is improved greatly. Moreover, any prior information about the motion can be applied in the model to get an improved performance of angular motion estimation.

### 2.1. GF-IMU Fixed Accelerometers Configurations

**Figure 1.**
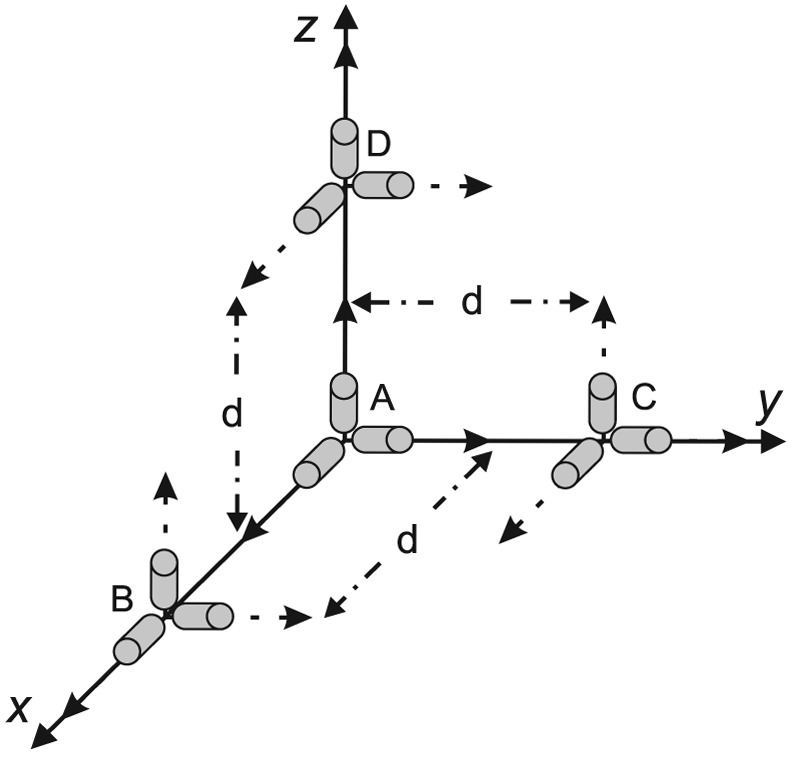
A configuration of multiple distributed tri-axial accelerometers.

The configuration shown in Figure 1, has four rigidly accelerometer triads, symbolized as A, B, C and D. We focus on configurations consisting of twelve mono-axial accelerometers that follow the rules listed by Zappa [8].

Mainly, we consider this configuration because a minimum of twelve accelerometers are needed to determine angular velocity magnitude and direction (algebraic sign cannot be determined uniquely). The most amount of the angular motion information, which is the AIV composed of the nine angular terms shown in Equations (1)–(3) can be extracted from this configuration. For more information about this special GF-IMU, we refer the reader to [5].

(1) $$\begin{array}{c} {\dot{\omega }}_{x}=\left(a_{z}^{C}-a_{z}^{A}-a_{y}^{D}+a_{y}^{A}\right)/2d\\ {\dot{\omega }}_{y}=\left(a_{x}^{D}-a_{x}^{A}-a_{z}^{B}+a_{z}^{A}\right)/2d\\ {\dot{\omega }}_{z}=\left(a_{y}^{B}-a_{y}^{A}-a_{x}^{C}+a_{x}^{A}\right)/2d \end{array}$$

(2) $$\begin{array}{c} \omega_{x}\omega_{y}=\left(a_{y}^{B}-a_{y}^{A}+a_{x}^{C}-a_{x}^{A}\right)/2d\\ \omega_{x}\omega_{z}=\left(a_{z}^{B}-a_{z}^{A}+a_{x}^{D}-a_{x}^{A}\right)/2d\\ \omega_{y}\omega_{z}=\left(a_{z}^{C}-a_{z}^{A}+a_{y}^{D}-a_{y}^{A}\right)/2d \end{array}$$

(3) $$\begin{array}{c} \omega_{x}^{2}=\left(a_{x}^{B}-a_{x}^{A}-a_{y}^{C}+a_{y}^{A}-a_{z}^{D}+a_{z}^{A}\right)/2d\\ \omega_{y}^{2}=\left(a_{y}^{C}-a_{y}^{A}-a_{x}^{B}+a_{x}^{A}-a_{z}^{D}+a_{z}^{A}\right)/2d\\ \omega_{z}^{2}=\left(a_{z}^{D}-a_{z}^{A}-a_{x}^{B}+a_{x}^{A}-a_{y}^{C}+a_{y}^{A}\right)/2d \end{array}$$

## 3. Dynamic Models to Be Considered

In general, a good model is important to extract the maximum amount of information from the observation. We will utilize the proper dynamic models in the angular motion estimation in the GF-IMU. We focus on the dynamic models used for maneuvering target tracking surveyed in [9]. Tracking theory dynamic models are used previously to model the angular acceleration evolution in time [10,11]. This work can be considered as a spatial motion extension to the planar motion case given in [11]. Assuming a certain type of motion the dynamic model can be formulated based on that assumption. The constant angular velocity model makes no use of the measured angular acceleration measurements so such a model is not a candidate for consideration in our work. Hence, we consider models in which the angular acceleration of the target is the descriptor of a target maneuver and modeled as a random process. Next, we describe the dynamic models that can be used for the Kalman filter process update.

### 3.1. Wiener-Process Angular Acceleration Model

This model assumes that the angular acceleration is a Wiener process, or more generally and precisely, the angular acceleration is a process with independent increments, which is not necessarily a Wiener process. This model is referred as a constant angular acceleration model (CAA) or a nearly constant angular acceleration model. It can be considered as a special case of a Gauss-Markov process. This model makes the angular acceleration a process with an increasing variance:

(4) $$\dot{\alpha }(t)=w(t)$$

The discrete-time form is given as:

(5) $$\alpha_{k}=\alpha_{k-1}+w_{k-1}$$

Since we have time uncorrelated noise, the corresponding state space representation of the Wiener sequence of angular acceleration vector combined with the angular velocity vector is given as:

(6) $$\left[\begin{array}{c} {\underline{\omega }}_{k}\\ {\underline{\alpha }}_{k} \end{array}\right]=\left[\begin{array}{cc} I_{3\times 3} & \Delta tI_{3\times 3}\\ 0_{3\times 3} & I_{3\times 3} \end{array}\right]\;\left[\begin{array}{c} \omega_{k-1}\\ \alpha_{k-1} \end{array}\right]+\left[\begin{array}{c} \Delta tI_{3\times 3}\\ I_{3\times 3} \end{array}\right]{\underline{w}}_{k-1}$$

### 3.2. First-Order Markov Model (Singer Angular Acceleration Model)

This model was initially used for modeling linear acceleration [12] and was lately used for angular acceleration modeling, as given in [10]. It has much wider coverage than constant angular velocity or constant angular acceleration models. The Singer model can be adjusted using the specifications of the accelerometers used. The Singer model assumes that the target acceleration is a zero-mean stationary first-order Markov process. The time evolution of the angular acceleration in continuous time is written as:

(7) $$\dot{\alpha }=-\beta \alpha +w$$

where *w* is a zero-mean white noise and *β* is the reciprocal of the time constant (or reciprocal of correlation time). The discrete form of this process is given as:

(8) $$\alpha_{k}=e^{-\beta \Delta t}\alpha_{k-1}+\sigma_{\alpha }\sqrt{1-e^{-2\beta \Delta t}}u_{k-1}$$

where *u_i_* is a sample generated from a Gaussian random number with a unit variance and *σ_a_* is the steady-state variance. Since the correlation time 1/*β* is much larger than sampling time Δ*t*, the following approximation is used:

(9) $$e^{-\beta \Delta t}\approx 1-\beta \Delta t$$

Considering a first-order linearization for the exponential term in Equations (9), the process can be approximated as:

(10) αk=(1−βΔt)αk−1+2βΔtσαuk−1^

The autocorrelation function Ψ*_α_* is exponentially decaying and given as:

(11) $$\Psi_{\alpha }=E\{\alpha (t+\tau )\alpha (t)\}=\sigma_{\alpha }^{2}e^{-\beta \left|\tau \right|}$$

The corresponding state space representation of the Wiener sequence angular acceleration model in 3D motion including angular velocity vector is given as:

(12) $$\begin{array}{l} \left[\begin{array}{c} {\underline{\omega }}_{k}\\ {\underline{\alpha }}_{k} \end{array}\right]=\left[\begin{array}{cc} I_{3\times 3} & \Delta tI_{3\times 3}\\ 0_{3\times 3} & (1-\beta \Delta t)I_{3\times 3} \end{array}\right]\;\left[\begin{array}{c} \omega_{k-1}\\ \alpha_{k-1} \end{array}\right]+\left[\begin{array}{c} \Delta tI_{3\times 3}\\ I_{3\times 3} \end{array}\right]{\underline{w}}_{k-1}\\ {\underline{w}}_{k-1}=\sqrt{2\beta \Delta t}\sigma_{\alpha }{\underline{u}}_{k-1} \end{array}$$

The variance is selected according to the ternary-uniform mixture as suggested in [10].

### 3.3. Other Models to Be Considered

Angular jerk, which is the derivative of the angular acceleration, can be used in the same way as that of the angular acceleration based models. Using angular jerk based models increases the dimension of the state space vector which increases the computational load.

## 4. Sensor Error Model and GF-IMU Calibration

In this section, we give a simple error model of the accelerometer which considers the bias only. The section ends with a review of simple calibration procedure which fits the GF-IMU.

### 4.1. The Accelerometer Error Model

Each accelerometer measurement is assumed to be corrupted by a bias *b_a_* error component and a continuous white noise error component *w_acc_*. The white noise usually has the unit of g/✓Hz, where *g* is the gravity, or its equivalent derivatives. All accelerometers are assumed to have a common upper bound for the noise variance and bias instability. The discrete-time white noise depends on the square root of the sampling time. The accelerometer measurement is modeled as:

(13) $$\tilde{a}=a+b_{a}+w_{\text{acc}}\sqrt{\Delta t}/\Delta t$$

The variance of the discrete-time noise component *R_disc._* of each measurement of the acceleration is:

(14) Rdisc^=E{wacc2}/Δt=Racc/Δt

Every drifting accelerometer bias has the unit of *g* or its equivalent derivatives and is modeled as a random process driven by white noise. The previously described Markov model is used often to model the bias or we can use the following simple model of random walk:

(15) $${\dot{b}}_{a}=w_{ba}$$

In discrete-time the random walk bias model is given as:

(16) $$b_{ak}=b_{ak-1}+b_{ba,k-1}$$

### 4.2. The Measured AIV

Using the accelerometer's error model shown in Equation (13), we rewrite the measured AIV with inherited accelerometers' errors based on Equations (1)–(3) as:

(17) $$\begin{array}{l} {\dot{\tilde{\omega }}}_{x}={\dot{\omega }}_{x}+b_{1}+W_{1}\\ \vdots \\ {\tilde{\omega }}_{z}^{2}=\omega_{z}^{2}+b_{9}+w_{9} \end{array}$$

where *b*_1_…*b*_9_ represent the new bias parameters and *w*_1_…*w*_9_ represent the noise errors.

### 4.3. Calibration and Initial Bias Estimation in a GF-IMU

In our setup, we adjust the separation distance manually to be unique for the three distributed triads with common orientation for all triads. Every accelerometer triad needs to be calibrated for three types of errors which are misalignment, scale factor and bias errors. Examples for the accelerometer triad calibration procedures can be found in [13,14]. The scale factor and misalignment parameters are contained in a three-by-three matrix and in our example they are calibrated only one time since they vary little with temperature change. The adopted calibration procedure is described with detailed equations in [5]. Any remaining bias parameters in the accelerometers results in a biased AIV. Once the IMU is detected in reset position, the AIV will be due to accelerometers' bias and hence its bias can be captured. Keeping the GF-IMU in a static position means all angular information terms should be almost zero because the quadratic terms due to Earth rotation are extremely small for small separation distance. Hence, we find the initial value for nine bias parameters in the AIV. In case of using the simple model without appending the bias parameters, the calibration process can compensate for the bias parameters in the AIV.

## 5. An EKF Solution Using the Singer Model with Appended Bias Parameters

Though we have twelve accelerometers and this means we have twelve unknown bias parameters, we are interested in estimating the resulting nine bias parameters *b*_1_…*b*_9_ in the AIV given in Equation (17). The state vector is composed of the angular velocity vector, the angular acceleration vector and the nine bias parameters given as:

(18) $$\begin{array}{l} \underline{x}={\left[\begin{array}{cccc} {\underline{\omega }}^{T} & {\underline{\alpha }}^{T} & {\underline{b}}_{\alpha }^{T} & {\underline{b}}_{\omega^{2}}^{T} \end{array}\right]}^{T},\underline{\omega }={\left[\begin{array}{ccc} \omega_{x} & \omega_{y} & \omega_{z} \end{array}\right]}^{T},\underline{\alpha }={\left[\begin{array}{ccc} {\dot{\omega }}_{x} & {\dot{\omega }}_{y} & {\dot{\omega }}_{z} \end{array}\right]}^{T}\\ {\underline{b}}_{\alpha }={\left[\begin{array}{ccc} b_{1} & b_{2} & b_{3} \end{array}\right]}^{T},{\underline{b}}_{\omega^{2}}={\left[\begin{array}{llllll} b_{4} & b_{5} & b_{6} & b_{7} & b_{8} & b_{9} \end{array}\right]}^{T} \end{array}$$

### 4.4. Initialization

The initial state vector can be set as:

(19) $${\underline{\hat{x}}}_{0}^{+}=E\{{\underline{x}}_{0}\}$$

The initial estimation error covariance is given as:

(20) $$P_{0}^{+}=E\{({\underline{x}}_{0}-{\underline{\hat{x}}}_{0}^{+}){\left({\underline{x}}_{0}-{\underline{\hat{x}}}_{0}^{+}\right)}^{T}\}$$

### 4.5. Prediction

Based on the previously described motion dynamic Equation (13) and the accelerometer bias Equation (16), we can write the discrete-time space model as:

(21) $$\begin{array}{l} {\left[\begin{array}{c} \underline{\omega }\\ \underline{\alpha }\\ {\underline{b}}_{\alpha }\\ {\underline{b}}_{\omega^{2}} \end{array}\right]}_{k}=\left[\begin{array}{cccc} I_{3\times 3} & \Delta tI_{3\times 3} & 0 & 0\\ 0 & (1-\beta \Delta t)I_{3\times 3} & 0 & 0\\ 0 & 0 & I_{3\times 3} & 0\\ 0 & 0 & 0 & I_{6\times 6} \end{array}\right]\;{\left[\begin{array}{c} \underline{\omega }\\ \underline{\alpha }\\ {\underline{b}}_{\alpha }\\ {\underline{b}}_{\omega^{2}} \end{array}\right]}_{k-1}+\left[\begin{array}{ccc} \Delta tI_{3\times 3} & 0 & 0\\ I_{3\times 3} & 0 & 0\\ 0 & I_{3\times 3} & 0\\ 0 & 0 & I_{6\times 6} \end{array}\right]\;{\left[\begin{array}{c} {\underline{w}}_{\alpha }\\ {\underline{w}}_{b_{a}}\\ {\underline{w}}_{b_{\omega^{2}}} \end{array}\right]}_{k-1}\\ {\underline{x}}_{k}=F_{k-1}{\underline{x}}_{k-1}+G_{k-1}{\underline{w}}_{k-1} \end{array}$$

The process covariance is computed as:

(22) $$Q_{k-1}=G\sum_{k-1}G^{T},\sum_{k-1}=E\{{\underline{w}}_{k-1}{\underline{w}}_{k-1}^{T}\}$$

The *a priori* estimation error covariance is updated as:

(23) $$P_{k}^{-}=F_{k-1}P_{k-1}^{+}F_{k-1}^{T}+Q_{k-1}$$

The *a priori* state estimate is predicted as:

(24) $${\underline{\hat{x}}}_{k}^{-}=F_{k-1}{\underline{\hat{x}}}_{k-1}^{+}$$

### 4.6. Measurement Update

The measurement vector is composed of the angular acceleration vector and six quadratic terms of angular velocity combined with the nine bias parameters is given as:

(25) $${\underline{h}}_{k}={\left[\begin{array}{ccccccccc} x_{4} & x_{5} & x_{6} & x_{1}x_{2} & x_{1}x_{3} & x_{2}x_{3} & x_{1}^{2} & x_{2}^{2} & x_{3}^{2} \end{array}\right]}_{k}^{T}+{\left[\begin{array}{ccccccccc} x_{7} & x_{8} & x_{9} & x_{10} & x_{11} & x_{12} & x_{13} & x_{14} & x_{15} \end{array}\right]}_{k}^{T}+{\underline{v}}_{k}$$

The measurement Jacobian matrix is computed as:

(26) $$H_{k}={\frac{\partial {\underline{h}}_{k}(\underline{x},\underline{v})}{\partial \underline{x}}|}_{x={\hat{x}}_{k}^{-}}={\left[\begin{array}{cccc} 0_{3\times 3} & I_{3\times 3} & I_{3\times 3} & 0_{3\times 6}\\ HH^{T} & 0_{6\times 3} & 0_{6\times 3} & I_{6\times 6} \end{array}\right]}_{x={\hat{x}}_{k}^{-}},HH\left[\begin{array}{cccccc} x_{2} & x_{3} & 0 & 2x_{1} & 0 & 0\\ x_{1} & 0 & x_{3} & 0 & 2x_{2} & 0\\ 0 & x_{1} & x_{2} & 0 & 0 & 2x_{3} \end{array}\right]$$

The measurement error covariance matrix is computed as:

(27) Rk=[RaMMTRω2],Rω2=Rdisc^d2[11/41/41/20−1/21/411/40−1/201/41/410001/2003/20−10−1/2003/2−1/2−1/200−1−1/23/2]Rα=Rdisc^d2[1−1/4−1/4−1/41−1/4−1/4−1/41],M=Rdisc^d2[−1/41/40−1/2−1/21/21/40−1/41/20−1/20−1/41/401/20]

The Kalman gain is updated as documented in literature e.g., Simon [15]:

(28) $$K_{k}=(P_{k}^{-}H_{k}^{T}){\left(H_{k}P_{k}^{-}H_{k}^{T}+R_{k}\right)}^{-1}$$

The *a posteriori* state estimate is updated as:

(29) $${\underline{\hat{x}}}_{k}^{+}={\underline{\hat{x}}}_{k}^{-}+K_{k}({\underline{y}}_{k}-{\underline{h}}_{k}({\underline{\hat{x}}}_{k}^{-},\underline{0}))$$

The *a posteriori* estimation error covariance can be updated as:

(30) $$P_{k}^{+}=P_{k}^{-}-K_{k}(H_{k}P_{k}^{-})$$

## 5. Observability Analysis

Using the dynamic model gives us the possibility to have all the bias parameters in the resulting angular terms observable under some conditions. In this section, we determine under which conditions is the state space observable. First, we remove the noise in this observability analysis which leaves us with the simpler homogeneous state-space system given in continuous-time as:

(31) $$\begin{array}{l} \underline{\dot{x}}=\underline{f}(\underline{x})=A\underline{x}\\ \underline{y}=\underline{h}(\underline{x})\\ A=\left[\begin{array}{ccc} 0_{3\times 3} & I_{3\times 3} & 0_{3\times 9}\\ 0_{12\times 3} & 0_{12\times 3} & 0_{12\times 9} \end{array}\right] \end{array}$$

where *A* is based on the CAA model and *h*(*x*) is the measurement function as described previously. We follow the local observability test based on Lie derivatives [16] in a similar way to its use in [11] for the one-dimensional angular motion. We compute *L*, which denotes the set of all finite linear combinations of Lie derivatives of the measurement vector with respect to *f*(*x*) for various values of constant input. For the *i*th row scalar measurement *h_i_* of the measurement vector, the Lie derivative of a scalar measurement is defined as:

(32) Lf(hi)=∂hi∂x_^f_(x_)

The zero-order Lie derivative of the measurement is the measurement itself, *i.e.*:

(33) $$L_{f}^{0}(h_{i})=h_{i}$$

Higher order Lie derivatives are computed as:

(34) $$L_{f}^{k}(h_{i})=h_{i}^{k}$$

For our model, which has *n* states, the higher order Lie derivatives starting from the third derivative are entirely zero vectors as shown next:

(35) $$h=\left[\begin{array}{c} x_{4}+x_{7}\\ x_{5}+x_{8}\\ x_{5}+x_{9}\\ x_{1}x_{2}+x_{10}\\ x_{1}x_{3}+x_{11}\\ x_{2}x_{3}+x_{12}\\ x_{1}^{2}+x_{13}\\ x_{2}^{2}+x_{14}\\ x_{3}^{2}+x_{15} \end{array}\right],\underline{\dot{h}}=\left[\begin{array}{c} 0\\ 0\\ 0\\ x_{2}x_{4}+x_{1}x_{5}\\ x_{3}x_{4}+x_{1}x_{6}\\ x_{3}x_{5}+x_{2}x_{6}\\ 2x_{1}x_{4}\\ 2x_{5}x_{2}\\ 2x_{3}x_{6} \end{array}\right],\ddot{\underline{h}}=\left[\begin{array}{c} 0\\ 0\\ 0\\ 2x_{4}x_{5}\\ 2x_{4}x_{6}\\ 2x_{5}x_{6}\\ 2x_{4}^{2}\\ 2x_{5}^{2}\\ 2x_{6}^{2} \end{array}\right]\ddot{\underline{h}}=\left[\begin{array}{c} 0\\ 0\\ 0\\ 0\\ 0\\ 0\\ 0\\ 0\\ 0 \end{array}\right],\ldots ,{\underline{h}}^{n-1}=\left[\begin{array}{c} 0\\ 0\\ 0\\ 0\\ 0\\ 0\\ 0\\ 0\\ 0 \end{array}\right]$$

The system is observable if the observability matrix *O*, which is defined next, has a rank equal to *n*:

(36) $$\begin{array}{l} O(x_{0},u)=\left[\begin{array}{c} dL_{f}^{0}(h_{1})\\ \vdots \\ dL_{f}^{0}(h_{p})\\ \vdots \\ dL_{f}^{n-1}(h_{1})\\ \vdots \\ dL_{f}^{n-1}(h_{p}) \end{array}\right]\\ dL_{f}^{k}(h_{i})=\frac{\partial L_{f}^{k}(h_{i})}{\partial \underline{x}} \end{array}$$

where *p* is the number of measurements. In our case, we have nine measurements and fifteen states so the dimension of the observability matrix is 135 × 15. After removing the zero rows, we get the following reduced observability matrix containing the independent rows:

(37) Ored^=[O11I9×9O2106×9O3106×9],O11=[03×3I3×3O21106×3],O211=[x2x10x30x10x3x22x10002x20002x3]O21=[x5x40x2x10x60x4x30x10x6x50x3x22x4002x10002x5002x20002x6002x3],O31=[0002x52x400002x602x400002x62x50004x40000004x50000004x6]

## 6. An EKF Solution Using the Singer Model without Appending Bias Parameters

Without appending bias parameters, the state vector is reduced to the angular velocity and the angular acceleration vectors and it is given as:

(38) $$\underline{x}={\left[\begin{array}{cc} {\underline{\omega }}^{T} & {\underline{\alpha }}^{T} \end{array}\right]}^{T},\underline{\omega }={\left[\begin{array}{ccc} \omega_{x} & \omega_{y} & \omega_{z} \end{array}\right]}^{T},\underline{\alpha }={\left[\begin{array}{ccc} {\dot{\omega }}_{x} & {\dot{\omega }}_{y} & {\dot{\omega }}_{z} \end{array}\right]}^{T}$$

The reduced state vector is clearly observable because the quadratic angular terms can solve for the angular velocity as shown in [8] and the angular acceleration terms are directly measurable so observability analysis in this case is not necessary.

### 6.1. Initialization

The initial state vector and initial estimation error covariance are assigned in a similar way as given in Equations (19) and (20).

### 6.2. Prediction

Based on the previously described dynamic model we can write the discrete-time space model as:

(39) $$\left[\begin{array}{c} {\underline{\omega }}_{k}\\ {\underline{\alpha }}_{k} \end{array}\right]=\left[\begin{array}{cc} I_{3\times 3} & \Delta tI_{3\times 3}\\ 0_{3\times 3} & (1-\beta \Delta t)I_{3\times 3} \end{array}\right]\;\left[\begin{array}{c} \omega_{k-1}\\ \alpha_{k-1} \end{array}\right]+\left[\begin{array}{c} \Delta tI_{3\times 3}\\ I_{3\times 3} \end{array}\right]{\underline{w}}_{k-1}$$

The *a priori* estimation error covariance and state estimate are predicted in a similar way to Equations (22) and (23).

### 6.3. Measurement Update

The AIV, which is composed of the angular acceleration vector and six quadratic terms of angular velocity, is given as:

(40) $${\underline{h}}_{k}={\left[\begin{array}{ccccccccc} x_{4} & x_{5} & x_{6} & x_{1}x_{2} & x_{1}x_{3} & x_{2}x_{3} & x_{1}^{2} & x_{2}^{2} & x_{3}^{2} \end{array}\right]}_{k}^{T}+{\underline{v}}_{k}$$

The measurement Jacobian matrix is computed as:

(41) $$H_{k}={\frac{\partial {\underline{h}}_{k}(\underline{x},\underline{v})}{\partial \underline{x}}|}_{x={\hat{x}}_{k}^{-}}={\left[\begin{array}{cc} 0_{3\times 3} & I_{3\times 3}\\ HH^{T} & 0_{6\times 3} \end{array}\right]}_{x={\hat{x}}_{k}^{-}},HH=\left[\begin{array}{cccccc} x_{2} & x_{3} & 0 & 2x_{1} & 0 & 0\\ x_{1} & 0 & x_{3} & 0 & 2x_{2} & 0\\ 0 & x_{1} & x_{2} & 0 & 0 & 2x_{3} \end{array}\right]$$

The Kalman gain, measurement error covariance matrix and the *a posteriori* state estimate are computed in a similar way to Equations (28)–(30).

## 7. Simulation Results for the Augmented Model

In this section, we give simulation results for a sinusoidal trajectory which is considered often in literature [7,10,11]. Moreover, such a trajectory satisfies the observability condition of a non-zero angular acceleration vector.

### 7.1. Trajectory Profile and Parameters Setting

This scenario of motion is a two-dimensional harmonic angular oscillation. Three-dimensional harmonic angular oscillation is considered as a coning motion, however, the GF-IMU system responds better in this case because of having all the AIV terms as non-zero, which increases observability. The mathematical description of the angular motion is given as:

(42) $$\underline{\omega }\left(t\right)=\omega_{m}\sin\left(2\pi ft\right){\left[\begin{array}{ccc} 1 & 1 & 0 \end{array}\right]}^{T}$$

(43) $$\underline{\alpha }\left(t\right)=2\pi f\omega_{m}\cos\left(2\pi ft\right){\left[\begin{array}{ccc} 1 & 1 & 0 \end{array}\right]}^{T}$$

For a practical value of the accelerometer's noise and bias levels, we consider the specifications of the accelerometers manufactured by Analog Devices. We want to have a portable IMU so we choose the separation distance to be 0.4 *m*. Duration time *T* of 20 s was enough to get stable results with sampling time of 0.01 s.

**Figure 2.**
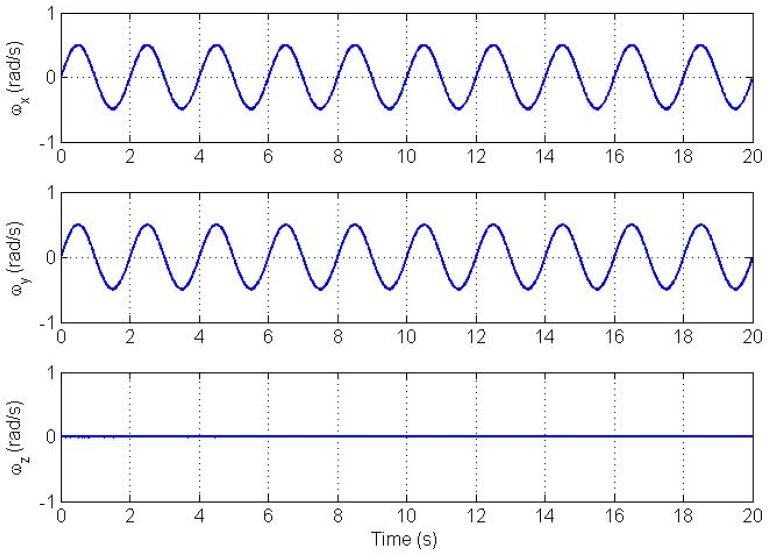
Angular velocity trajectory profile.

**Figure 3.**
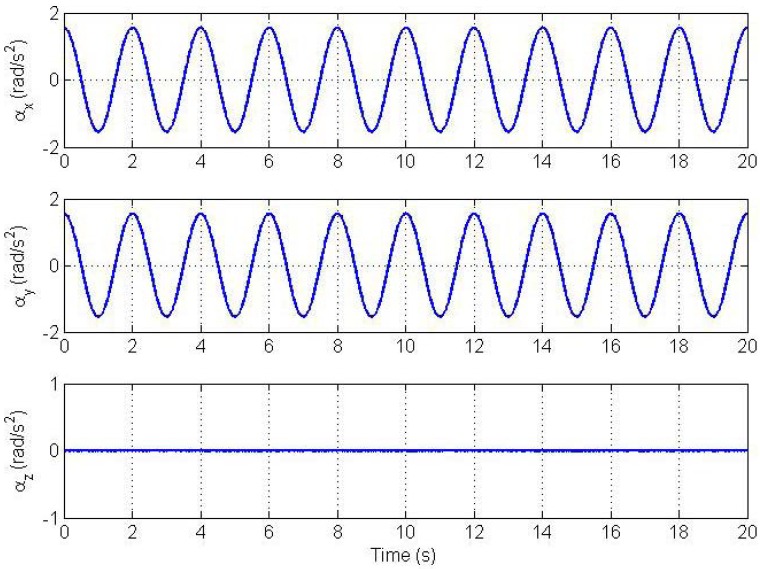
Angular acceleration trajectory profile.

The state vector is initialized properly around the true state with initial state error of 5% of the true value. The bias values were selected randomly from a distribution with one standard deviation of 2400 μg as shown in Table 1. Figures 2 and 3 show plots of the angular velocity and the angular acceleration profiles respectively.

### 7.2. Results and Analysis

**Figure 4.**
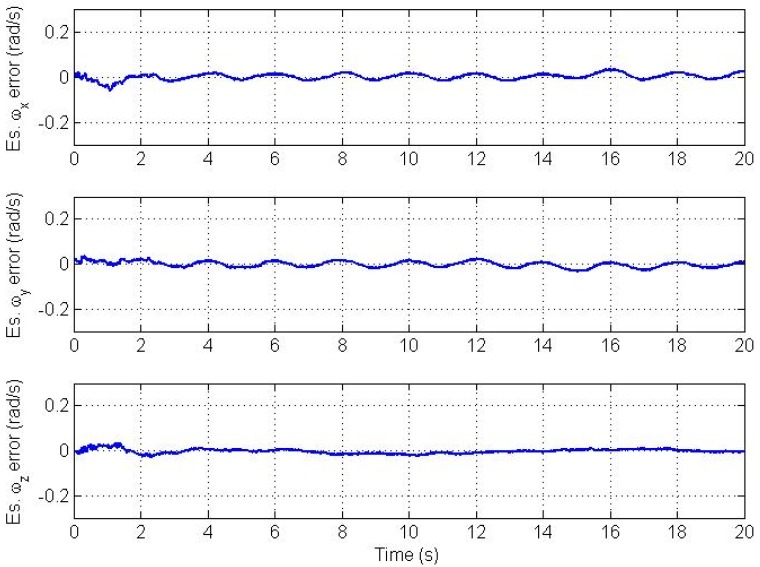
Angular velocity vector estimation errors.

**Figure 5.**
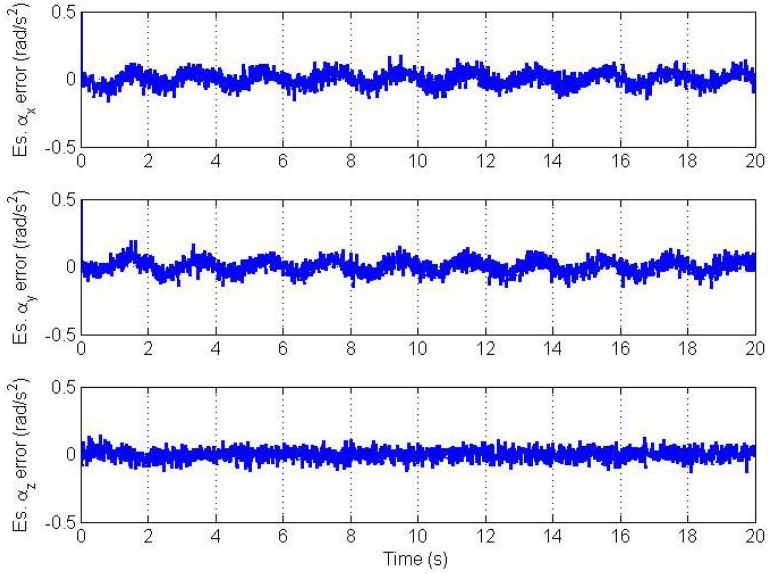
Angular acceleration vector estimation errors.

Errors in the estimated angular velocity and angular acceleration vectors are plotted in Figures 4 and 5, respectively.

**Figure 6.**
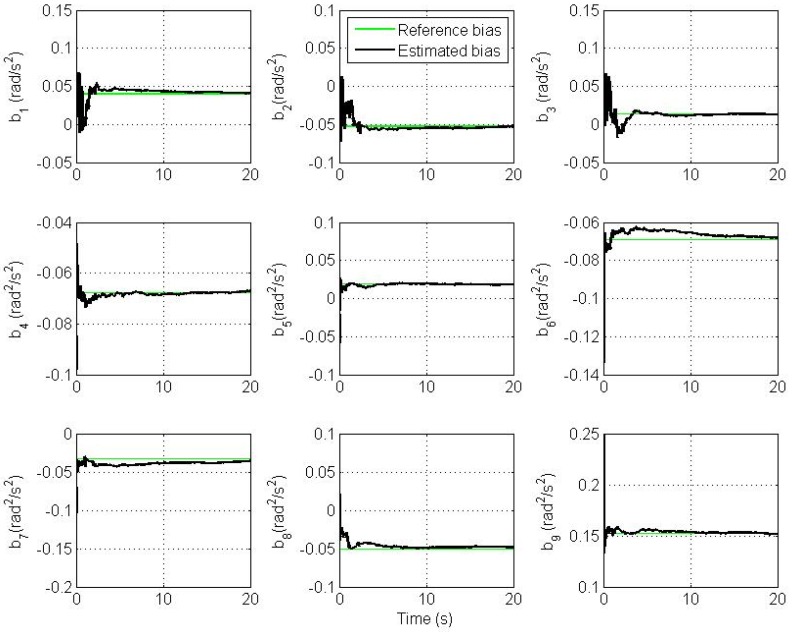
Estimated and reference bias parameters in the AIV.

Clearly, we see a good convergence to the true angular velocity and angular acceleration components. However, there is a small oscillation in the *x* and y components of the angular velocity and angular acceleration. This is because we model the angular acceleration as a random process driven by white noise, while the true trajectory is a sinusoidal one. From simulations, the magnitude of this swing increases as the frequency of oscillation of the trajectory increases and *vice versa* if the trajectory's frequency of oscillation decreases. Therefore, the CAA and the Markov models are not suitable for highly dynamic oscillations. The plots of estimation errors in the nine bias parameters are shown in Figure 6.

The plots show the convergence of all estimated bias parameters in AIV to their exact value with small steady state error. Moreover, from extensive simulations we find that reducing the noise error level of accelerometers gives a smoother and a faster convergence of bias parameters.

### 7.3. Effect of Improper Initialization

**Figure 7.**
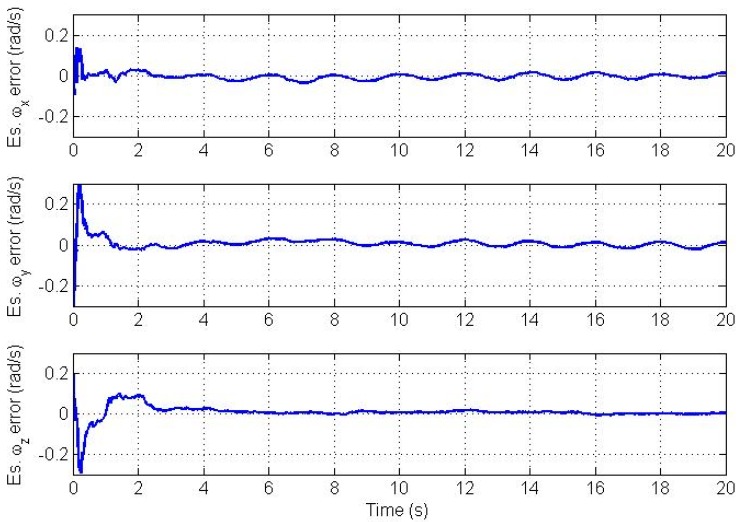
Angular velocity vector estimation errors for improper initialization.

**Figure 8.**
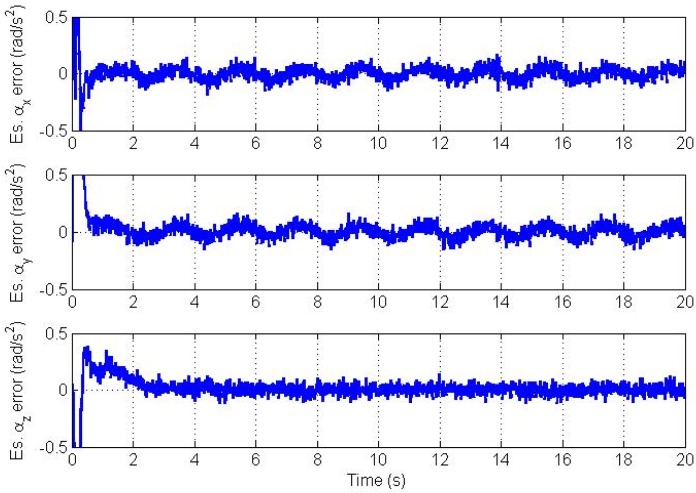
Angular acceleration vector estimation errors for improper initialization.

It is well known that proper initialization of the state vector for the EKF, which implies linearization, is important to avoid filter divergence. However, the filter can tolerate a limited level of initialization error if the nonlinearity is not high. Since there is no *a priori* information available about bias parameters in AIV from calibration, we initialize the bias vector with zeros. Errors in the estimated angular velocity and angular acceleration vectors for the improper initialization case are plotted in Figures 7 and 8 respectively. Simulations show that the filter converges without problems but it takes longer time under the same values of bias stability which was used before.

## 8. Simulation Results for Non-Augmented Model

In this part of the simulations, we consider the same trajectory described previously, but with bias parameters non-appended to the state vector which means we simply ignored them. For this reduced model, we can find the criteria for ignoring bias parameters based on accelerometers specifications of bias and noise errors given in Table 2.

At relatively high sampling rates (e.g., 0.01 s or more), the magnitude of the error due to white noise is about 10 times the magnitude of error due to remaining bias for a tactical grade accelerometer. Consequently, for this sampling rate, the noise error dominates the bias error in this accelerometer category and for this scenario the EKF model works without a big difference from the one without bias. Hence, ignoring the bias and approximating the error as white noise error model can be justified considering that the Kalman filter will tolerate such a small remaining bias error.

### 8.1. Trajectory Profile and Parameter Setting

We repeat the previously used trajectory profile with the same settings except for the noise and bias levels which are set to *w_acc._* = 100 μg/✓Hz and the bias selected randomly from distribution with one standard deviation of 100 *μg*. The chosen accelerometer specifications satisfy the criteria that the error is dominated by white noise error at the selected sampling rate of 0.01 s.

### 8.2. Results and Analysis

**Figure 9.**
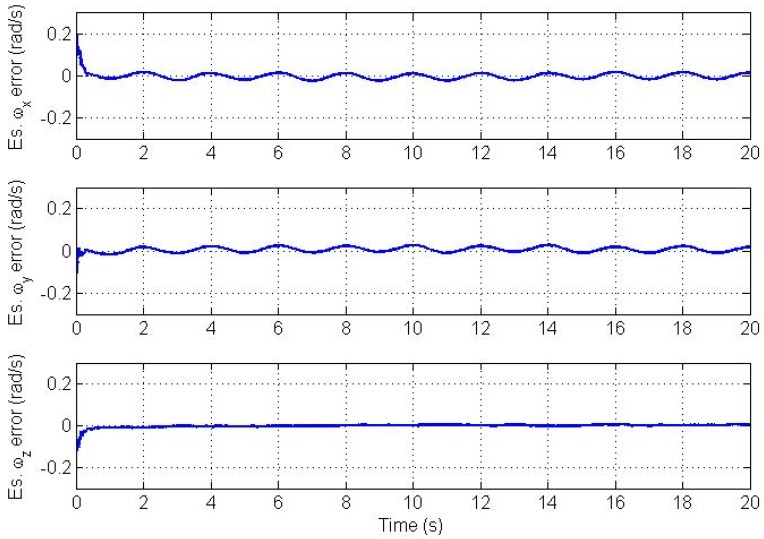
Angular velocity vector estimation errors for the reduced model.

**Figure 10.**
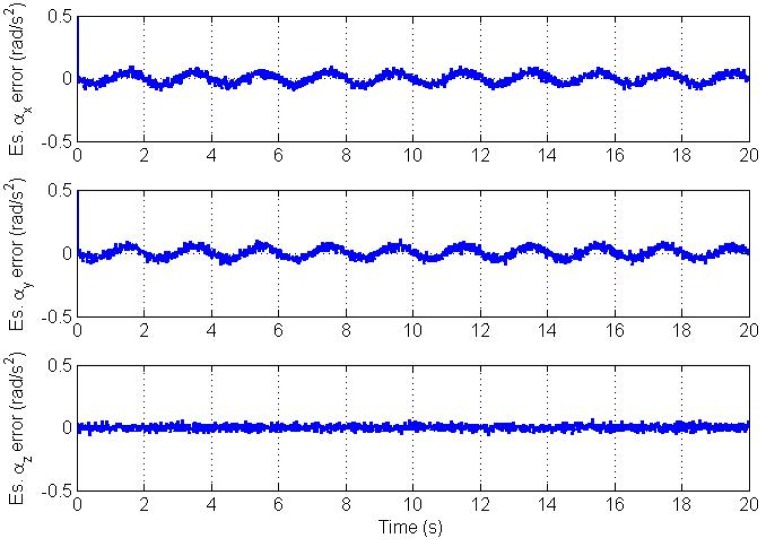
Angular acceleration vector estimation errors for the reduced model.

First, the execution time for this model was much smaller than that of the previous model, which has bias parameters appended, because we have a much simpler state model. Using a reduced model implies reducing computational load remarkably. The plots of errors in the estimated angular velocity and the estimated angular accelerations are shown in Figures 9 and 10, respectively. From both figures, we see a fast convergence to the true profile of angular velocity and angular acceleration. Again, we see a small oscillation in the *x* and *y* components of the angular velocity and the angular acceleration, which was explained previously in Subsection 8.2.

**Figure 11.**
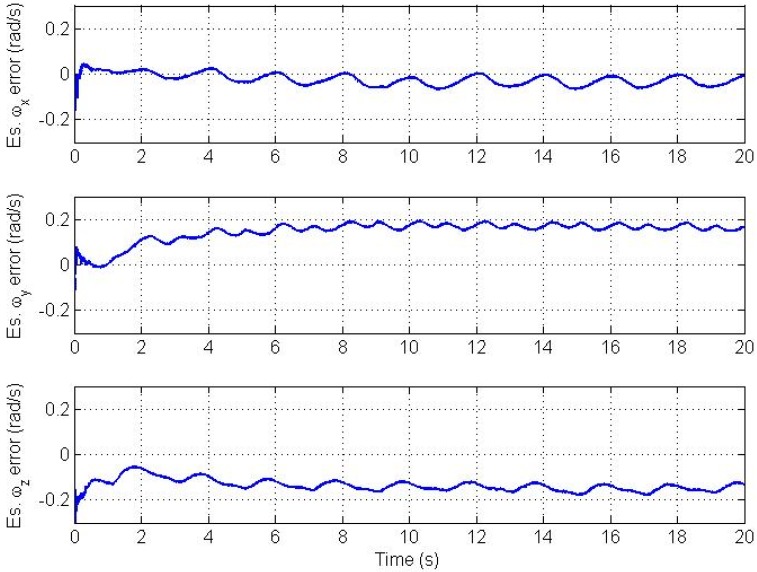
Angular velocity vector estimation errors for the reduced model with large AIV bias values.

To see the effect of ignoring higher bias values in the AIV, we repeated the simulation with the same parameter values as used in subsection 8.1 and created a plot of the estimated angular velocity vector. Such values of parameters do not meet the criteria that accelerometer's error is dominated by white noise error and hence this results in a biased estimate of the angular velocity vector as shown in Figure 11.

## 9. Conclusions

We have presented a novel solution for estimating the spatial angular motion and bias parameters in a GF-IMU utilizing the dynamic models. The integration scheme is performed using an EKF. Observability analysis for the augmented model shows that the state space model is observable whenever the angular acceleration vector has non-zero magnitude. Simulation results shows that the filter can estimate the angular motion and bias parameters in the AIV for proper and improper initialization. Moreover in case of using tactical grade accelerometers or better, the error is dominated by noise error and hence the model can possibly be reduced to include only angular motion terms without degrading performance. Further research can be done to estimate the remaining bias parameters in the accelerometers.

## Figures and Tables

**Table 1.** Numerical values of the simulation parameters.

**Parameter (Unit)**	***ω_m_* (rad/s)**	***f* (Hz)**	***w_acc_* (g/✓Hz)**	***bias* (g)**	***_d_*_(m)_**
Value	0.4112	0.5	200 *μ*	2400 *μ*	0.4

**Table 2.** Accelerometer Categories.

**Performance Parameter**	**Consumer**	**Automotive**	**Tactical**	**Navigation**
Noise Floor VRW (μg/✓Hz)	2000	1000	100–400	5–10
Bias Stability (μg)	2400	1200	50–500	5–10
